# Association between the onset timing of suicidal ideation and the means of severe suicide attempts in patients with schizophrenia

**DOI:** 10.1002/pcn5.70150

**Published:** 2025-07-06

**Authors:** Hitomi Ito, Kentaro Fukumoto, Kotaro Otsuka

**Affiliations:** ^1^ Department of Neuropsychiatry, School of Medicine Iwate Medical University Iwate Japan; ^2^ Morioka City Medical Association Affiliated Morioka Nursing College Morioka Japan; ^3^ Department of Disaster and Community Psychiatry and Department of Neuropsychiatry Iwate Medical University Morioka Japan

**Keywords:** emergency, means of suicide, schizophrenia, suicidal ideation, suicide attempt

## Abstract

**Aim:**

To investigate the timing of the onset of suicidal ideation in patients with schizophrenia, the severity of suicide attempts, and associated factors.

**Methods:**

A total of 273 patients with schizophrenia visiting the emergency department for suicide attempts between January 2003 and December 2021 were included in this study. The participants were categorized into two groups: the same‐day (SD) group and the prior‐to‐same‐day (PSD) group, based on the timing of the onset of suicidal ideation. Data were collected on patient demographics and psychiatric symptoms observed at the time of visit. Additionally, information regarding suicidal motives and the means used for suicide attempts was analyzed to explore the relationship between the timing of suicidal ideation, the severity of suicide attempt means, and associated factors.

**Results:**

The PSD group was significantly more likely to select severe means of suicide attempts compared to the SD group (*p* = 0.03). In the SD group, a strong positive association was identified between the use of severe suicide means and suicidal motives linked to hallucinations and delusions (odds ratio [OR] = 2.01, 95% confidence interval [CI] 1.01–4.03, *p* = 0.049). Conversely, in the PSD group, a negative association was observed between the history of suicide attempts within the past year and selection of severe means (OR = 0.32, 95% CI 0.12–0.86, *p* = 0.023).

**Conclusion:**

The timing of suicidal ideation significantly influences the severity of suicide attempt means. These findings provide critical insights for enhancing suicide risk assessment and intervention strategies in patients with schizophrenia.

## Graphical abstract

This study investigated the timing of suicidal ideation onset, the severity of suicide attempts, and related factors in schizophrenia patients.

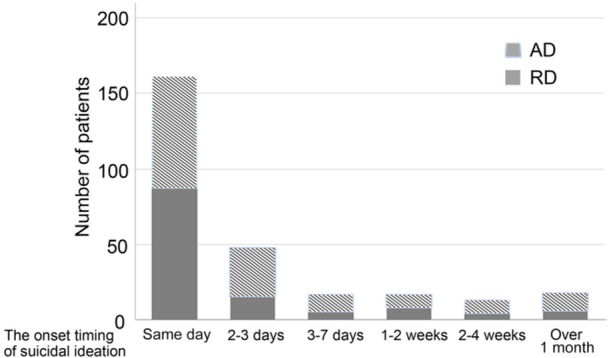

## INTRODUCTION

According to the World Health Organization (WHO), it is estimated that over 720,000 individuals globally die by suicide each year, with approximately 20 suicide attempts occurring for every completed suicide.^1^ Suicide ranks among the top 20 leading causes of death, and reducing suicide‐related mortality remains a key priority for the WHO. In Japan, 21,837 suicides were recorded in 2023, with health‐related issues identified as the most prevalent motive for suicide. Notably, mental illnesses account for a significant proportion of these suicides.^2^

Schizophrenia, a common mental disorder, is associated with a reduction in life expectancy by an estimated 10 to 20 years. Suicide is a major contributor to this reduced lifespan, with a lifetime prevalence of suicidal ideation reported at 19% and a suicide rate of 1.5% among individuals with schizophrenia, figures markedly higher than those in the general population.^3^, ^4^, ^5^ Previous research has identified various risk factors for suicidal ideation in schizophrenia, including younger age, male gender, higher premorbid intelligence quotient, minimal cognitive impairment, chronic disease progression, younger age at onset, prolonged untreated illness, prior suicide attempts, co‐occurring depressive symptoms, auditory hallucinations, insomnia, hopelessness, and a history of childhood maltreatment.^6^, ^7^, ^8^, ^9^, ^10^, ^11^

Suicidal ideation is characterized by persistent thoughts and preoccupations with suicide, often accompanied by intense emotional distress. Conversely, suicide attempts entail actual engagement in self‐harming behaviors with the intent to end one's life. Suicide attempts are not only significant predictors of eventual suicide but also indicators of severe psychological distress.^12^ The progression from suicidal ideation to a suicide attempt is influenced by a complex interplay of factors, including anxiety, impulsivity, and underlying psychiatric disorders. Suicidal ideation, however, is frequently an independent phenomenon and does not necessarily culminate in suicide attempts.^13^ For instance, a study of older adults revealed that only 4.8% experienced suicidal thoughts in the 2 weeks preceding a suicide attempt.^14^ Furthermore, meta‐analyses in adults have demonstrated that the predictive value of suicidal ideation for subsequent suicide is modest, with limited positive predictive value.^15^ While many cases of suicidal ideation resolve spontaneously, this is not universally observed.^13^ Some individuals transition rapidly from the onset of suicidal ideation to a suicide attempt, whereas others may attempt suicide following a prolonged period of persistent ideation.

Otsuka et al. examined the characteristics of psychiatric patients who arrived at emergency care after suicide attempts, focusing on the duration between suicidal ideation and the attempt. Their findings indicated that a longer interval between the onset of suicidal ideation and the suicide attempt correlated with an increased likelihood of employing more severe means.^13^ Nevertheless, no studies have specifically explored this temporal relationship in individuals with schizophrenia, therefore the aim of the present study was to investigate the association between the onset timing of suicidal ideation and the means of severe suicide attempts in patients with schizophrenia.

## METHODS

### Participants

Between January 1, 2003, and December 31, 2021, a total of 26,244 patients visited the psychiatric emergency department of Iwate Medical University Hospital. Among them, 5817 patients were diagnosed with schizophrenia based on the International Statistical Classification of Diseases and Related Health Problems, 10th Revision.^16^ Patients whose primary diagnosis was schizophrenia were included even if they had comorbid conditions. Among this group of patients, 546 individuals with confirmed suicide attempts were identified. The assessment of diagnosis, suicide attempts, suicidal ideation, and the timing of ideation was conducted by emergency psychiatrists or attending psychiatrists at the hospital under the supervision of a senior psychiatrist (a Designated Psychiatrist under the Act on Mental Health and Welfare in Japan). Suicide attempts were determined using Kishi's classification,^17^ which includes the following criteria: (1) a self‐reported suicide attempt, (2) the presence of a suicide note or verbal suicide threat, (3) a witness to the suicide attempt, (4) confirmation by judicial authorities or autopsy, and (5) verification by a physician. Patients were classified as suicides if they were confirmed deceased on arrival or died within 24 h of hospital admission. Retrospective analysis was conducted on the medical records of 273 eligible patients, focusing on the means of suicide attempts and the timing of suicidal ideation. Based on the timing of suicidal ideation, patients were categorized into two groups: the same‐day (SD) group, where suicidal ideation and the suicide attempt occurred on the same day, and the prior‐to‐same‐day (PSD) group, where suicidal ideation occurred at least 24 h before the suicide attempt.

### Survey and assessment items

Data were obtained from the medical records of the patients regarding demographic and clinical characteristics, including gender, age, highest level of educational attainment, employment status, co‐habitation status, status of psychiatric outpatient care, medication adherence, past episodes of mood disorders, lifetime and recent (past year) history of suicide attempts, onset of current suicidal ideation, as well as the motivation and means of suicide attempts. This information was collected from the patients themselves, their family members, or acquaintances. A psychiatrist who evaluated the patients during their emergency room visits assessed their psychiatric symptoms at the time of presentation using the Japanese version of the Brief Psychiatric Rating Scale (BPRS)^18^ and evaluated their overall functioning with the Global Assessment Scale (GAS).^19^ The BPRS comprises 18 items, each scored on a 7‐point scale ranging from 1 (no symptoms) to 7 (most severe), with higher scores indicating greater symptom severity. In contrast, the GAS is a unidimensional scale where higher scores denote better overall functioning. Additionally, the Holmes and Rahe Social Readjustment Rating Scale^20^ was employed to evaluate life events preceding the suicide attempt. Life Change Unit (LCU) scores derived from this scale, reflecting the intensity of stressful life events, were also incorporated into the analysis, with higher scores corresponding to greater levels of stress. All evaluations were conducted by psychiatrists affiliated with Iwate Medical University Hospital. The severity of suicide attempt means was classified according to Asukai's classification,^21^ which classifies patients into two categories—absolute danger (AD) group and relative danger (RD) group—based on the physical severity of the suicide attempt (Table S1). This classification is based on the assessment of life‐threatening risk and physical severity. This study included patients for whom data on the motives and means of suicide attempts, as well as psychiatric symptoms observed at the time of presentation, were available. As some patients exhibited multiple motives, means, and psychiatric symptoms, these overlapping factors were accounted for in the analysis.

### Statistical analysis

Statistical analyses were conducted using IBM SPSS Statistics, version 30.0 (IBM). The association between the onset of suicidal ideation and the severity of suicide attempts was evaluated using the chi‐square test. Patient demographics, psychiatric symptoms at the time of the emergency department visit, and motives for suicide attempts were compared between the two groups. Furthermore, to identify factors associated with the means of severe suicide attempts in the SD and PSD groups, patients were categorized into AD and RD groups, and comparisons were made regarding patient demographics, psychiatric symptoms, and motives for suicide attempts. Continuous variables were analyzed using the Mann–Whitney *U* test, while categorical variables were assessed using the chi‐square test or Fisher's exact test. For the BPRS, GAS, and LCU scales, cases with missing data were excluded due to incomplete datasets. Binomial logistic regression analysis was subsequently employed to identify factors associated with severe suicide attempt means (AD). Explanatory variables included age, gender, and items that demonstrated significant differences between the AD and RD groups. Adjusted odds ratios and their corresponding 95% confidence intervals were calculated. All statistical tests were two‐tailed, with a significance threshold set at *p* < 0.05.

### Ethics approval

This study received approval from the Ethics Committee of the Iwate Medical University School of Medicine (MH2022‐163) and was conducted in strict adherence to ethical standards. An opt‐out mechanism was employed, allowing potential participants to decline involvement. Specifically, a detailed explanatory document outlining the study's purpose and procedures was published on the institution's website, along with clear instructions for individuals to formally register their refusal within a designated timeframe.

## RESULTS

### The timing of the onset of suicidal ideation and the severity of suicide attempts

A total of 273 patients (mean age 35.9 ± 13.3 years) were analyzed, comprising 74 males (mean age 39.1 ± 12.0 years) and 199 females (mean age 34.8 ± 13.6 years). Among them, 149 patients (54.6%) employed severe means in their suicide attempts **(**Table S2). The most frequently used means was drug overdose, reported by 151 patients (55.3%), followed by attempts involving bladed or piercing instruments in 56 patients (20.5%), and jumping from a height in 32 patients (11.7%) (Table S3).

The timing of suicidal ideation was categorized into six distinct intervals: the day of the suicide attempt, 2–3 days, 3–7 days, 1–2 weeks, 2–4 weeks, and more than 1 month prior. The proportion of patients employing severe means (AD group) was analyzed. Suicidal ideation occurred on the day of the attempt in 160 patients, accounting for 58.6% of the total. Among these, 74 patients (46.3%) belonged to the AD group. Conversely, 33 patients (68.8%) from the 48 whose suicidal ideation occurred 2–3 days prior to the attempt were classified in the AD group. Similarly, patients with suicidal ideation more than 3 days prior to the attempt exhibited a higher proportion in the AD group compared to the RD group (Figure S1). A significant association was observed between the timing of suicidal ideation and the use of severe means (χ²[5] = 12.3, *p* < 0.05).

### Patient backgrounds, symptoms, and motivations by onset of suicidal ideation

In studies involving psychiatric patients who presented to emergency psychiatric services, it has been suggested that the background characteristics differ between those who experienced suicidal ideation on the day of their suicide attempt and those who had suicidal ideation prior to that day.^13^ Accordingly, in the present study, patients who experienced suicidal ideation on the day of their suicide attempt (*n* = 160) were classified as the same‐day (SD) group, while those who exhibited suicidal ideation prior to the day of the suicide attempt (*n* = 113) were categorized as the pre‐same‐day (PSD) group. The demographic and clinical characteristics, psychiatric symptoms at the time of emergency department visit, and motives for suicide were compared between the two groups (Tables S2 and S4). The SD group was significantly older than the PSD group (*p* = 0.01) and demonstrated a lower incidence of more severe suicide attempts (*p* = 0.001). However, there were no significant differences between the groups in the severity of psychiatric symptoms, as measured by the BPRS and GAS scores. Similarly, no differences were observed in the types of psychiatric symptoms present at the emergency department or in the motives underlying the suicide attempts (Table S4).

### Factors associated with the means of severe suicide attempts

Given the observed association between the timing of suicidal ideation and the severity of suicide attempts, we investigated factors related to the means of severe suicide attempts according to the timing of suicidal ideation to conduct a more detailed analysis. Initially, patients in the SD group were divided into two subgroups: the AD group (*n* = 74) and the RD group (*n* = 86). We compared their demographic characteristics, psychiatric symptoms at the time of the visit to the emergency department, and motives for the suicide attempt (Tables 1 and 2). No significant differences were observed in demographic characteristics; however, the AD group demonstrated a higher proportion of patients with disturbances of thought at the time of the emergency department visit (*p* = 0.024) and a greater prevalence of suicide attempts driven by hallucinatory delusions (*p* = 0.023). Binomial logistic regression analysis revealed a significant positive association between the AD group and suicide attempts motivated by hallucinatory delusions (odds ratio [OR] = 2.01, 95% confidence interval [CI] 1.01–4.03, *p* = 0.049) (Table 3). Patients in the PSD group were similarly divided into the AD group (*n* = 75) and the RD group (*n* = 38), and the same analyses were performed as for the SD group (Tables 1 and 2 **)**. Unlike the SD group, no differences in psychiatric symptoms or motives for the suicide attempt at the time of the emergency department visit were identified. However, the proportion of patients with a history of suicide attempts within the past year was significantly lower in the AD group compared to the RD group (*p* = 0.048). Binomial logistic regression analysis further indicated a significant negative association between the AD group and a history of suicide attempts within one year (OR = 0.32, 95% CI 0.12–0.86, *p* = 0.023) (Table 3).

**Table 1:** Demographic data of the AD and RD groups.

Item	Total					SD group					PSD group				
	(*n* = 273)					(*n* = 160)					(*n* = 113)				
	AD group		RD group			AD group		RD group			AD group		RD group		
	(*n* = 149)		(*n* = 124)		*p* value	(*n* = 74)		(*n* = 86)		*p* value	(*n* = 75)		(*n* = 38)		*p* value
Age (years)	36.5	(12.7)	35.3	(14.0)	0.36	35.3	(12.7)	33.5	(13.4)	0.29	37.7	(12.6)	39.4	(14.7)	0.54
Gender (male)	46	(30.9)	28	(22.6)	0.14	23	(31.1)	19	(22.1)	0.21	23	(30.7)	9	(23.7)	0.51
Highest level of education															
Less than junior high school	2	(1.3)	0	(0)	0.42	1	(1.4)	0	(0)	0.12	1	(1.3)	0	(0)	0.38
Junior high school	29	(19.5)	21	(16.9)		13	(17.6)	16	(18.6)		16	(21.3)	5	(13.2)	
High school	69	(46.3)	66	(53.2)		33	(44.6)	51	(59.3)		36	(48.0)	15	(39.5)	
Junior college, vocational school, or university	20	(13.4)	11	(8.9)		12	(16.2)	5	(5.8)		8	(10.7)	6	(15.8)	
Unknown	29	(19.5)	26	(21.0)		15	(20.3)	14	(16.3)		14	(18.7)	12	(31.6)	
Employment status (employed)	31	(20.8)	24	(19.4)	0.88	15	(20.3)	19	(22.1)	0.85	16	(21.3)	5	(13.2)	0.44
Co‐habitation status (cohabitant(s) present)	117	(78.5)	102	(82.3)	0.45	57	(77.0)	72	(83.7)	0.32	60	(80.0)	30	(78.9)	1.0
Status of psychiatric outpatient care (ongoing treatment)	82	(55.0)	72	(58.1)	0.63	43	(58.1)	54	(62.8)	0.63	39	(52.0)	18	(47.4)	0.69
Previous history of depressive episodes (one or more)	84	(56.4)	59	(47.6)	0.18	43	(58.1)	41	(47.7)	0.21	41	(54.7)	18	(47.4)	0.55
History of suicide attempts (present)	66	(44.3)	62	(50.0)	0.39	34	(45.9)	45	(52.3)	0.43	32	(42.7)	17	(44.7)	0.84
History of suicide attempts within 1 year (present)	28	(18.8)	41	(31.1)	<0.001*	17	(23.0)	29	(33.7)	0.16	11	(14.7)	12	(31.6)	0.048*
Medication adherence (maintained)	54	(36.2)	37	(29.8)	0.30	26	(35.1)	24	(27.9)	0.39	28	(37.3)	13	(34.2)	0.84
GAS	27.4	(14.8)	31.9	(17.1)	0.05	26.8	(13.8)	32.2	(17.7)	0.08	28.0	(15.9)	31.2	(15.8)	0.37
BPRS total score	27.1	(16.7)	25.0	(17.1)	0.21	26.0	(17.6)	24.2	(17.9)	0.36	28.3	(15.8)	26.9	(14.9)	0.76
LCU	21.0	(31.1)	19.0	(29.0)	0.85	17.6	(31.6)	18.4	(24.4)	0.36	24.1	(30.6)	20.0	(37.1)	0.31

*Note*: Values in parentheses are percentages or standard deviations. Age, GAS, BPRS, and LCU are presented with their respective standard deviations in parentheses, while all other values in parentheses represent percentages. Complete demographic information was not collected for all patients (GAS: total *n* = 256, SD group *n* = 153, PSD group *n* = 103; BPRS total score: total *n* = 252, SD group *n* = 151, RD group *n* = 101; LCU: total *n* = 221, SD group *n* = 124, PSD group *n* = 97).

Abbreviations: AD group, absolute danger group; BPRS, brief psychiatric rating scale; GAS, global assessment scale; LCU, life change unit; PSD group, prior‐to‐same‐day group; RD group, relative danger group; SD group, same‐day group.

* *p* < 0.05 was defined as significant.

**Table 2:** Psychiatric symptoms and causes of suicidal ideation in the AD and RD groups.

Item	Total					SD group					PSD group				
	(*n* = 273)					(*n* = 160)					(*n* = 113)				
	AD group		RD group			AD group		RD group			AD group		RD group		
	(*n* = 149)		(*n* = 124)		*p* value	(*n* = 74)		(*n* = 86)		*p* value	(*n* = 75)		(*n* = 38)		*p* value
Psychiatric symptoms at emergency department visit															
Disturbance of consciousness	69	(46.3)	66	(53.2)	0.28	31	(41.9)	46	(53.5)	0.16	38	(50.7)	20	(52.6)	1.0
Disturbance of self‐awareness^#^	8	(5.4)	3	(2.4)	0.36	4	(5.4)	2	(2.3)	0.42	4	(5.3)	1	(2.6)	0.66
Disturbance of perception	47	(31.5)	32	(25.8)	0.35	25	(33.8)	19	(22.1)	0.11	22	(29.3)	13	(34.2)	0.67
Disturbance of thought	71	(47.7)	47	(37.9)	0.11	38	(51.4)	28	(32.6)	0.024*	33	(44.0)	19	(50.0)	0.11
Disturbance of motivation	42	(28.2)	34	(27.4)	1.0	22	(29.7)	23	(26.7)	0.73	20	(26.7)	11	(28.9)	0.83
Disturbance of emotion	110	(73.8)	82	(66.1)	0.18	56	(75.7)	55	(64.0)	0.12	54	(72.0)	27	(71.1)	1.0
Sleep disturbance^#^	3	(2.0)	2	(1.6)	1.0	2	(2.7)	1	(1.2)	0.60	1	(1.3)	1	(2.6)	1.0
Impairment of memory^#^	1	(0.7)	1	(0.8)	1.0	0	(0)	1	(1.2)	1.0	1	(1.3)	0	(0)	1.0
Side effects of psychotropic drugs^#^	4	(2.7)	1	(0.8)	0.38	2	(2.7)	1	(1.2)	0.60	2	(2.7)	0	(0)	0.55
Causes of suicidal ideation															
Family or home	24	(16.1)	14	(11.3)	0.29	13	(17.6)	12	(14.0)	0.66	11	(14.7)	2	(5.3)	0.21
Financial problems^#^	5	(3.4)	4	(3.2)	1.0	1	(1.4)	4	(4.7)	0.37	4	(5.3)	0	(0)	0.30
Suffering due to illness	18	(12.1)	13	(10.5)	0.71	7	(9.5)	9	(10.5)	1.0	11	(14.7)	4.0	(10.5)	0.77
Hallucinations/delusions	70	(47.0)	41	(33.1)	0.026*	37	(50.0)	27	(31.4)	0.023*	33	(44.0)	14	(36.8)	0.55
Work/school^#^	4	(2.7)	5	(4.0)	0.74	3	(4.1)	4	(4.7)	1.0	1	(1.3)	1	(2.6)	1.0
Interpersonalrelationships	8	(5.4)	17	(13.7)	0.021*	4	(5.4)	12	(14.0)	0.11	4	(5.3)	5	(13.2)	0.16

*Note*: Values in parentheses are percentages. Since multiple items may apply to a single patient, the numbers for each category represent the total counts. Items marked with # were analyzed using Fisher's exact test, while all other items were analyzed using the chi‐square test.

Abbreviations: AD group, absolute danger group; BPRS, brief psychiatric rating scale; GAS, global assessment scale; LCU, life change index scale; PSD group, prior‐to‐same‐day group; RD group, relative danger group; SD group, same‐day group.

* *p* < 0.05 was defined as significant.

**Table 3:** Factors associated with severe suicide attempts in patients with schizophrenia.

(a) Factors associated with severe suicide attempts in the SD group						
Item	B	SE	Wald	Odds ratios	95% CI	*p*
Gender^a^	0.46	0.38	1.49	1.59	0.79–3.35	0.22
Age (years)	0.01	0.01	0.71	1.01	0.99–1.03	0.40
Psychiatric symptoms; disturbance of thought^b^	0.58	0.35	2.76	1.78	0.90–3.51	0.10
Causes of suicidal ideation; hallucinations/delusions^b^	0.70	0.35	3.89	2.01	1.01–4.03	0.049*

(b) Factors associated with severe suicide attempts in the PSD group						
Item	B	SE	Wald	Odd ratios	95% CI	*p*
Gender^a^	0.45	0.47	0.90	1.56	0.62–3.94	0.34
Age (years)	−0.02	0.02	1.36	0.98	0.95–1.01	0.98
History of suicide attempts within 1 year^b^	−1.14	0.50	5.16	0.32	0.12–0.86	0.023*

Abbreviations: CI, confidence interval; PSD group, prior‐to‐same‐day group; SD group, same‐day group; SE, standard error.

* *p* < 0.05 was defined as significant.

^a^ Female coded 0, male coded 1.

^b^ Absent coded 0, present coded 1.

## DISCUSSION

This study focused on the relationship between the timing of suicidal ideation and the severity of suicide attempts in schizophrenia. The findings revealed that individuals who experienced suicidal ideation prior to the day of their suicide attempt employed more severe means compared to those whose suicidal ideation emerged on the same day as the attempt. Analysis of suicidal ideation onset indicated that serious suicide attempts occurring on the same day were significantly associated with hallucinatory delusions. Conversely, patients who engaged in severe suicide attempts after experiencing suicidal ideation for 2 or more days demonstrated a significantly lower proportion of having a history of suicide attempts within the preceding year.

More than half of the patients who attempted suicide experienced an overdose. Previous studies, including those involving non‐schizophrenic patients, have indicated that individuals with suicidal ideation occurring on the same day as the suicide attempt were more likely to attempt suicide using drugs compared to those with suicidal ideation lasting for more than 1 day.^13^ In the current study, however, no significant difference was observed in the rate of drug‐related suicide attempts between the SD and PSD groups (data not shown). Interestingly, patients whose suicidal ideation emerged before the day of the suicide attempt were more likely to choose more severe means of suicide, a finding consistent with past reports. Notably, 46% of patients (Table S2) made a severe suicide attempt on the day their suicidal ideation emerged, a figure significantly higher than the 9.5% reported in previous studies across all psychiatric disorders, not limited to schizophrenia.^13^ Suicide attempts are not universally driven by the intention to die but can often represent an act of help‐seeking.^22^ Overdose has also been reported to be used with the intention of alleviating the fear of death.^23^ However, it is well known that schizophrenic patients find it difficult to engage in help‐seeking behaviors.^24^ This difficulty likely explains the relatively low number of patients using non‐severe means of suicide attempts in this study, resulting in a higher proportion of patients engaging in severe suicide attempts.

We analyzed variables commonly associated with suicidal ideation in schizophrenia, including younger age, male gender, depression, auditory hallucinations, and insomnia. Aside from the observation that patients in the SD group were younger than those in the PSD group, no significant associations were identified between specific psychiatric symptoms and the timing of the onset of suicidal ideation (Tables S2 and S4). Factors associated with the means of severe suicide attempts differed between the SD group and the PSD group. In the SD group, a significant association was observed between hallucinations and delusions as the motive for suicide and the choice of severe means for the attempt. Many studies have reported a strong association between hallucinations and delusions in schizophrenia and suicide.^25^, ^26^, ^27^ Consistent with these findings, the present study also found that the proportion of patients whose suicide attempts were associated with hallucinations or delusions was higher in the group that opted for severe means. In contrast, no significant difference was observed between the AD and RD groups in the PSD group (Table 2). These findings suggest that hallucinations and delusions contributing to suicide attempts may cause profound psychological distress in patients with schizophrenia. Gooding et al.^28^ reported that when positive symptoms such as hallucinations and delusions are associated with suicidal ideation, the increased risk of suicide is mediated not only by the presence of the symptoms themselves but also by the cognitive and emotional sequelae in response to these symptoms. This distress may lead individuals to engage in severe suicide attempts on the same day that suicidal ideation emerges because they are unable to suppress or delay these thoughts until the following day. Furthermore, such hallucinations and delusions may include imperative auditory hallucinations that directly instruct patients to attempt suicide, as well as self‐deprecating delusions that undermine their sense of self‐worth. Analysis incorporating these mediating factors remains an important subject for future research. In the PSD group, there was a negative correlation between a history of suicide attempts within the past year and the use of severe means in subsequent attempts. While a history of previous suicide attempts is a well‐recognized risk factor for suicide in schizophrenia,^6^, ^25^, ^29^ the results of this study suggest that it does not necessarily correlate with progression to more severe means of suicide attempts.

The findings of the present study underscore the importance of developing intervention strategies tailored to the timing of suicidal ideation in patients with schizophrenia. In the SD group, a significant association was observed between hallucinations or delusions and severe suicide attempts. Accordingly, in clinical settings, when positive symptoms are identified, it is essential to carefully assess their severity and, when appropriate, promptly adjust antipsychotic treatment to achieve adequate symptom control. In particular, when patients with insufficiently controlled positive symptoms report suicidal ideation, immediate intervention is warranted to prevent serious suicide attempts. It is crucial to ensure that rapid access to psychiatric evaluation is available, and, when necessary, psychiatric hospitalization should be considered as a part of the risk management strategy. Conversely, the findings from the PSD group suggest that even in the absence of a suicide attempt within the past year, patients with schizophrenia who report suicidal ideation may still be at elevated risk of engaging in serious suicidal behavior. To address this risk, it is recommended that a continuous and comprehensive monitoring system be established, incorporating home visits, frequent outpatient follow‐up, and close collaboration with caregivers.

## LIMITATIONS

This study has several limitations. Primarily, it was conducted at a single institution, which may limit the generalizability of the findings. The value of this survey design should not be underestimated, considering that identifying and analyzing 273 patients with schizophrenia who have attempted suicide requires substantial time and resources, even in multicenter research. Another limitation of this study is that we were unable to collect information on the course of illness, such as age of onset, whether the illness was in an acute or chronic phase, and the duration of untreated illness, as well as cognitive function. These factors are considered to be associated with suicide attempts in schizophrenia.^6^, ^7^, ^8^, ^9^, ^10^, ^11^ Furthermore, although the type and dosage of psychotropic medications, as well as concomitant medications, may have an influence on both suicide attempts and the methods employed, these factors could not be examined in detail in the present study. These limitations highlight the need for further investigations to clarify the association between prescription patterns and suicidal behavior. Finally, the cross‐sectional design of this study limits causal inference and precludes the examination of the temporal dynamics of suicidal ideation. Future longitudinal research is warranted to elucidate how suicidal ideation in patients with schizophrenia progresses and fluctuates over time, particularly in relation to psychotic symptoms, treatment factors, and environmental stressors.

## CONCLUSION

This study underscores the clinical importance of incorporating the timing of suicidal ideation into suicide risk assessments in patients with schizophrenia. Our findings indicate that both the temporal pattern of suicidal ideation and the presence of psychotic symptoms may be associated with the severity of suicidal behavior. Integrating these factors into clinical evaluations may support the development of more effective, individualized intervention strategies. Further longitudinal research is warranted to elucidate the dynamic interplay between suicidal ideation, psychotic symptomatology, and the risk of severe suicide attempts over time.

## AUTHOR CONTRIBUTIONS

Kotaro Otsuka and Kentaro Fukumoto contributed to the conceptualization of the study. Hitomi Ito and Kentaro Fukumoto analyzed the data. Hitomi Ito wrote the original manuscript. All authors participated in discussion and revisions. All authors read and approved the final version of the manuscript.

## CONFLICT OF INTEREST STATEMENT

The authors declare no conflicts of interest.

## ETHICS APPROVAL STATEMENT

This study was approved by the Ethics Committee of Iwate Medical University School of Medicine (MH2022‐163).

## PATIENT CONSENT STATEMENT

In the survey, informed consent was not obtained from participants as an opt‐out approach was adopted. An explanatory document outlining the study's objectives and content was made available on the facility's website, accompanied by a procedure allowing participants to express their intention to decline participation within a specified period.

## CLINICAL TRIAL REGISTRATION

N/A.

## Supporting information

Sup Table.

figure1_0123.

supmat.

## Data Availability

N/A.
