# Supplementary material for: Association between the onset timing of suicidal ideation and the means of severe suicide attempts in patients with schizophrenia
Source: PCN Rep. 2025 Jul 6;4(3):e70150. doi: 10.1002/pcn5.70150 (PMC12230198; doi:10.1002/pcn5.70150)
Supplement: Supplementary file 1 — Sup Table. [file PCN5-4-e70150-s003.pdf]

**Supplementary Table 1. Asukai Classification for Suicide Attempters[21)]**

| Means of suicide | The conditions corresponding to the Absolutely Dangerous (AD) group |
|---|---|
| Overdose/Poison ingestion | Ingestion of a lethal dose of medication or the utilization of mechanical ventilation or dialysis |
| Jumping from a height | A height of four stories or more than 10 meters |
| Vehicle or train jumping | All cases |
| Bladed or piercing instruments | Injuries to major arteries and veins, critical nerves, the trachea, and vital internal organs |
| Self-immolation | Second-degree burns covering 30% or more of the body surface area,<br>third-degree burns affecting 10% or more, or airway burns. |
| Gaseous substances | Individuals presenting with impaired consciousness at the time of discovery |
| Suicidal drowning | Loss of consciousness or respiratory failure upon discovery |
| Hanging-induced asphyxiation | Individuals presenting with impaired consciousness at the time of discovery |

If any of the aforementioned conditions are met, the patient is categorized as belonging to the absolutely dangerous (AD) group; otherwise, the patient is classified as part of the relatively dangerous (RD) group.

**Supplementary Table 2. Demographics data**

| Item | Total | | SD group | | PSD group | | p value |
|---|---|---|---|---|---|---|---|
| | (n = 273) | | (n = 160) | | (n = 113) | | |
| Age (year) | 35.9 | (13.3) | 34.3 | (13.1) | 38.3 | (13.3) | 0.01* |
| Gender (male) | 74 | (27.1) | 42 | (26.3) | 32 | (28.3) | 0.78 |
| Highest level of education | | | | | | | |
| Less than a junior high school | 2 | (0.7) | 1 | (0.6) | 1 | (0.9) | |
| Junior high school | 50 | (18.3) | 29 | (18.1) | 21 | (18.6) | |
| High school | 135 | (49.5) | 84 | (52.5) | 51 | (45.1) | 0.78 |
| Junior colleges, vocational schools, or universities | 31 | (11.4) | 17 | (10.6) | 14 | (12.4) | |
| Unknown | 55 | (20.1) | 29 | (18.1) | 26 | (23.0) | |
| Employment status (employed) | 55 | (20.1) | 34 | (21.3) | 21 | (18.6) | 0.65 |
| Cohabitation status (cohabitant(s) present) | 219 | (80.2) | 129 | (80.6) | 90 | (79.6) | 0.88 |
| Status of psychiatric outpatient care (ongoing treatment) | 154 | (56.4) | 97 | (60.6) | 57 | (50.4) | 0.06 |
| Previous history of depressive episodes (one or more) | 143 | (52.4) | 84 | (52.5) | 59 | (52.2) | 1.0 |
| History of suicide attempts (present) | 128 | (46.9) | 79 | (49.4) | 49 | (43.4) | 0.39 |
| History of suicide attempts within 1 year (present) | 69 | (25.3) | 46 | (28.7) | 23 | (20.4) | 0.12 |
| Medication adherence (maintained) | 91 | (31.3) | 50 | (31.3) | 41 | (36.3) | 0.44 |
| GAS | 29.4 | (16.0) | 29.7 | (16.1) | 29.0 | (15.9) | 0.85 |
| BPRS Total Score | 26.2 | (16.9) | 25.1 | (17.7) | 27.8 | (15.4) | 0.05 |
| LCU | 20.1 | (30.1) | 18.0 | (27.9) | 22.8 | (32.7) | 0.29 |
| Severity of Suicide Attempt Means (AD group according to the Asukai classification) | 149 | (54.6) | 74 | (46.3) | 75 | (66.4) | 0.001* |

Values in parentheses are percentages or standard deviations.

SD group: Same-day group, PSD group: Prior-to-same-day group,AD group: absolutely dangerous group, GAS: Global assessment scale,

BPRS: Brief Psychiatric Rating Scale, LCU: Life Change Unit

Age, GAS, BPRS, and LCU are presented with their respective standard deviations in parentheses, while all other values in parentheses represent percentages.

Complete demographic information was not collected for all patients (GAS: Total n=256, SD group n=153, PSD group n=103; BPRS Total Score: Total n=252, SD group n=151, RD group n=101; LCU: Total n=221, SD group n=124, PSD group n=97).

*p <0.05 was defined as significant.

**Supplementary Table 3. Means of suicide**

| Means | Total (n = 273) | |
|---|---|---|
| Overdose | 151 | (55.3) |
| Poison ingestion | 20 | (7.3) |
| Jumping from a height | 32 | (11.7) |
| Vehicle or train jumping | 1 | (0.4) |
| Bladed or piercing instruments | 56 | (20.5) |
| Self-immolation | 3 | (1.1) |
| Gaseous substances | 9 | (3.3) |
| Suicidal drowning | 3 | (1.1) |
| Hanging-induced asphyxiation | 8 | (2.9) |

Values in parentheses are percentages.

Since multiple items may apply to a single patient, the numbers for each category represent the total counts.

**Supplementary Table 4. Psychiatric Symptoms and Causes of Suicidal Ideation**

| Item | Total | | SD group | | PSD group | | p value |
|---|---|---|---|---|---|---|---|
| | (n = 273) | | (n = 160) | | (n = 113) | | |
| Psychiatric Symptoms at Emergency Department Visit | | | | | | | |
| Disturbance of Consciousness | 135 | (49.5) | 77 | (48.1) | 58 | (51.3) | 0.63 |
| Disturbance of Self-Awareness# | 11 | (4.0) | 6 | (3.8) | 5 | (4.4) | 0.77 |
| Disturbance of Perception | 79 | (28.9) | 44 | (27.5) | 35 | (31.0) | 0.78 |
| Disturbance of Thought | 118 | (43.2) | 66 | (41.3) | 52 | (46.0) | 0.46 |
| Disturbance of Motivation | 76 | (27.8) | 45 | (28.1) | 31 | (27.4) | 1.0 |
| Disturbance of Emotion | 192 | (70.3) | 111 | (69.4) | 81 | (71.7) | 0.79 |
| Sleep disturbance# | 5 | (1.8) | 3 | (1.9) | 2 | (1.8) | 1.0 |
| Impairment of memory# | 2 | (0.7) | 1 | (0.6) | 1 | (0.9) | 1.0 |
| Side effects of psychotropic drugs# | 5 | (1.8) | 3 | (1.9) | 2 | (1.8) | 1.0 |
| Causes of Suicidal Ideation | | | | | | | |
| Family or Home | 38 | (13.9) | 25 | (15.6) | 13 | (11.5) | 0.38 |
| Financial Problems# | 9 | (3.3) | 5 | (3.1) | 3 | (3.5) | 1.0 |
| Suffering due to illness | 31 | (11.4) | 16 | (10.0) | 15 | (13.3) | 0.44 |
| Hallucinations / Delusions | 111 | (40.7) | 64 | (40.0) | 47 | (41.6) | 0.80 |
| Work / School# | 9 | (3.3) | 7 | (4.4) | 2 | (1.8) | 0.31 |
| Interpersonal Relationships | 25 | (16.0) | 16 | (10.0) | 9 | (8.0) | 0.67 |

Values in parentheses are percentages.

SD group: Same-day group, PSD group: Prior-to-same-day group

Since multiple items may apply to a single patient, the numbers for each category represent the total counts.

Items marked with # were analyzed using Fisher's exact test, while all other items were analyzed using the chi-square test.
