# Supplementary figures and images for: Association between the onset timing of suicidal ideation and the means of severe suicide attempts in patients with schizophrenia
Source: PCN Rep. 2025 Jul 6;4(3):e70150. doi: 10.1002/pcn5.70150 (PMC12230198; doi:10.1002/pcn5.70150)

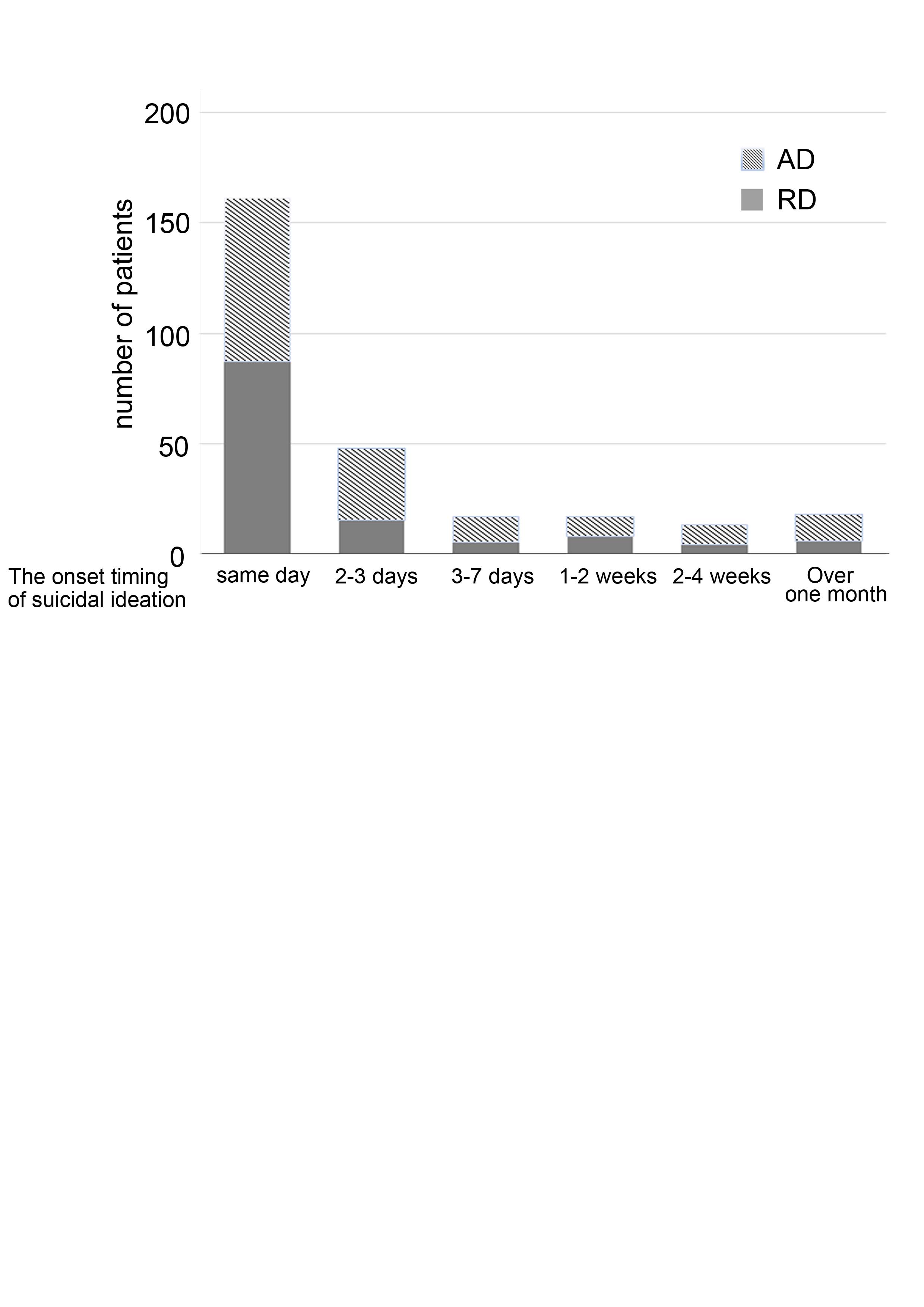

Supplement: Supplementary file 2 — figure1_0123. [file PCN5-4-e70150-s002.jpg]
