# Supplementary material for: Association between the onset timing of suicidal ideation and the means of severe suicide attempts in patients with schizophrenia
Source: PCN Rep. 2025 Jul 6;4(3):e70150. doi: 10.1002/pcn5.70150 (PMC12230198; doi:10.1002/pcn5.70150)
Supplement: Supplementary file 3 — supmat. [file PCN5-4-e70150-s001.docx]

**Supplementary Figure legends**

Supplementary Figure 1. Timing of Suicidal Ideation Onset and Severity of Suicide Attempts in Patients with Schizophrenia. The horizontal axis represents the time (in days) from the onset of suicidal ideation to the suicide attempt. The vertical axis indicates the number of patients. Shaded bars represent the absolutely dangerous (AD) group, and grey bars represent the relatively dangerous (RD) group.
